# Supplementary material for: Phylogeny analysis of whole protein-coding genes in metagenomic data detected an environmental gradient for the microbiota
Source: PLoS One. 2023 Feb 2;18(2):e0281288. doi: 10.1371/journal.pone.0281288 (PMC9894459; doi:10.1371/journal.pone.0281288)
Supplement: S1 Text — (DOCX) [file pone.0281288.s001.docx]

**Supporting information**

**S1 Text. Procedure for estimating the metagenomic distance in the MPASS method**

The MPASS method was developed from our previous method that was based on average similarities of whole genome sequences for the construction of phylogenomic trees [23]. For the metagenomic analysis, we modified the step that calculated genomic distances.

To calculate the metagenomic distance, we first compared two proteomes and obtained the best-matched protein pairs. We obtained the e-values for all the protein sequences by BLASTP searches (Exy_1_, Exy_2_, …, Exy_n_), where X is the query proteome and Y is the reference proteome. We also obtained e-values against the query sequences themselves (Exx_1_, Exx_2_, …, Exx_n_). Next, all the e-values of the Exx and Exy searches were log-transformed and multiplied by the *k-*tuple coverage (Cov) for each protein sequence ($Cov\times\log_{10} Exx$, $Cov\times\log_{10} Exy$). *K-*tuple coverages were obtained when the fastq reads were assembled by metaSPAdes [44]. Then, by averaging all the $Cov\times\log_{10} Exx$ and $Cov\times\log_{10} Exy$ values, we obtained the average (Av) values (Av_Exx_, Av_Exy_), respectively. We used the average values and the cut-off value for the BLAST calculation (Log_10_Bco) to calculate the similarity (m) between two metagenomes as

$m=\frac{{Av}_{Exy}-\log_{10} Bco}{{Av}_{Exx}-\log_{10} Bco}$.

Theoretically, m = 1 means that two metagenomes are identical and m = 0 means that two metagenomes have no similarity. The relative difference T between the m derived from real metagenomic data and the m for two identical metagenomes (m = 1) was calculated as

$T=\frac{1-m}{1+m}$.

Finally, we transformed the proteomic distance T to obtain the distance between genome data S for the nucleotide-based unit according to our previous study [23] as follows:

$S=const1\times e^{(const2\times T)}$.

In the MPASS method, we calculated the constants (const1 and const2) from the regression analysis using values T and the percentage of Poisson-corrected substitution rates of 16S rRNA sequences, and determined const1 and const2 as 4.142 and 2.824, respectively (S1 Fig.). Therefore, the nucleotide-based metagenomic distance S can be estimated as

$S=4.142\times e^{(2.824\times T)}$.
