# Supplementary figures and images for: Phylogeny analysis of whole protein-coding genes in metagenomic data detected an environmental gradient for the microbiota
Source: PLoS One. 2023 Feb 2;18(2):e0281288. doi: 10.1371/journal.pone.0281288 (PMC9894459; doi:10.1371/journal.pone.0281288)

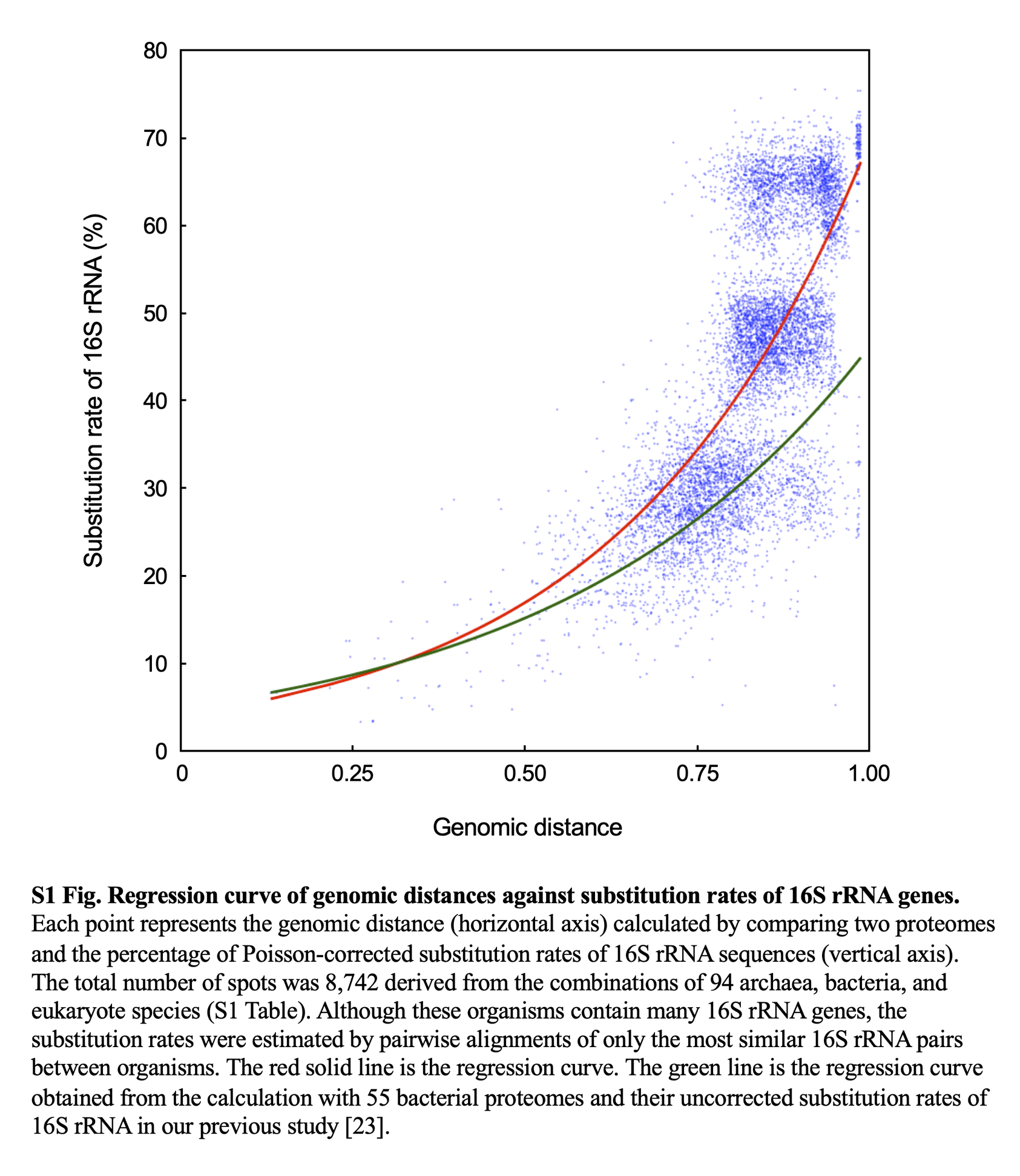

Supplement: S1 Fig — (TIF) [file pone.0281288.s002.tif]
