# Supplementary material for: Phylogeny analysis of whole protein-coding genes in metagenomic data detected an environmental gradient for the microbiota
Source: PLoS One. 2023 Feb 2;18(2):e0281288. doi: 10.1371/journal.pone.0281288 (PMC9894459; doi:10.1371/journal.pone.0281288)
Supplement: Attachment — Submitted filename: Response to Reviewers_SSatoh.docx [file pone.0281288.s008.docx]

Jan 8, 2023

Response to Reviewers

Thank you for your careful reading of our manuscript and valuable comments. We agreed with most of the comments by all reviewers and extensively revised our manuscript. The parts of the manuscript that have been revised are highlighted in red.

According to the comments, we improved our methodology by the application of a new calculation method, and provided the merged program code for almost all of the steps in MPASS processes and the additional Perl script for the automatic prefiltering of fastq files. We reconstructed all metagenomic trees and their related data with the improved program. We also provided the minimal data set for the results described in the manuscript as a file “S1File.zip”. Although almost all reconstructed metagenomic trees and their related data were identical to those of the previous version of the manuscript, minor corrections associated with these reconstructed data in the manuscript are highlighted in red. We also highlighted the slight descriptive errors and correction of the format of the manuscript in red. I hope you will satisfy our revision.

Soichirou Satoh

**Answers to the comment of Reviewer 2**

Thank you for your critical and careful reading. These comments are valuable for improving our manuscript and I greatly appreciate them. We have revised our manuscript according to your suggestions. I hope you will satisfy our revision. The followings are the answers to your comments.

**Comment;**

Line 383:

When calculating const1 and const2, the reviewer was surprised that the authors do not conduct any correction for multiple substitutions in the same site. Since uncorrected methods tend to underestimate genetic distance, especially for the comparison between distantly related taxa (See molecular evolution textbook), the authors must correct multiple substitutions. Estimating const1 and const2 without correcting multiple substitutions is nonsense.

**Answer to the comment;**

We fully agreed with your comment that correction for multiple substitutions in the same site is very important. As this suggestion, we improved our methodology by adopting the Poisson-corrected substitution rate for the calculation of the metagenomic distances, and reconstructed all metagenomic trees and their related data with this new calculation method. Although almost all reconstructed metagenomic trees reproduced the same topology as that of trees constructed with the previous MPASS program, we think that our method is improved by your suggestions.

**Comment;**

Line 402:

Ref. 26 does not say these taxa inhabit soils. Taxa choices are not adequate.

To my knowledge, some of them do not inhabit soils.

*Sulfolobus islandicus*: thermophilic archaea, habitat is hot springs

*Proteus mirabilis*: pathogen, habitat is mammal gut but can be found in polluted soil

*Nitrosospira multiformis*: habitat is soil. OK.

*Bacteroides fragilis*: a very famous human gut microbe

*Acidobacterium capsulatum*: habitat is soil. OK.

**Answer to the comment;**

We replaced the phrase “five soil bacteria species” with “five bacteria species”.

**Comment;**

Figure S1:

This figure does not indicate substitution rates but just sequence identities.

**Answer to the comment;**

We calculated the Poisson-corrected substitution rates between 16S rRNA genes of various organisms, and showed the relationship of them to distances from the comparison of metagenomes in Fig. S1.

**Comment;**

Provide the program code for MPASS. Otherwise other researchers cannot use MPASS. Maybe MPASS is a pipeline, so provide complete code is difficult. But at least the authors should provide some shell scripts that are merged some steps of MPASS processes in Fig. 1. These comments are only representative of methodologies. The reviewer has many other comments related to the metagenomic analysis results of this paper, but before the analysis results, using adequate methodology is necessary.

**Answer to the comment;**

We fully agreed with your comment that the availability of the MPASS program by other researchers is also very important. I also hope that this program is used by many researchers to study various metagenomes. According to this suggestion, we provided two Perl scripts. First, we provided automatic program code for the down-sampling, assembling, gene prediction, and calculation of the metagenomic distances in the MPASS pipeline. Second, we also provided another Perl script for the automatic prefiltering of multiple pairs of fastq files by using the Trimmomatic software. We uploaded these two scripts (MPASS.pl, FilteringFastq_for_MPASS.pl) and a refined version of the previous script MPASS_core.pl (only for the metagenomic distance calculation) on the GitHub website (https://github.com/s0sat/MPASS), and wrote this URL in the manuscript. By using MPASS.pl and FilteringFastq_for_MPASS.pl, the researchers can construct the metagenomic distance matrices from their paired-end fastq files or those downloaded from public databases, automatically.
